# An Important Ruling

**Published:** 1907-05

**Authors:** 


An Important Ruling.—Disinfection of Public Buildings Act Includes Theaters, Hotels, Restaurants, City Halls, Jails, Etc.—Will Issue New Rules.—Quite an important ruling has been made by Judge William E. Hawkins, First Assistant Attorney General, on the construction of the Act of 1903, requiring the disinfection of public buildings. The ruling was made at the request of State Health Officer Brumby, who is preparing a set of rules and regulations on the subject. A number of places or buildings are named by Judge Hawkins as coming within the provisions of the act, and it is given a liberal construction. The new rules and regulations will be ready within a short time. Dr. Brumby is determined to maintain at a high standard the sanitary condition of Texas, especially as the summer is rapidly approaching. The following is the full text of the ruling or opinion:

*William M. Brumby, M. D., State Health Officer, Capitol.*

DEAR SIR: We have your letter of recent date, in which you say:

"I intend issuing a circular relating to the disinfection of public buildings in the State, but before doing so would like to have an opinion from you upon the construction to be placed upon Section 1, Senate Bill No. 95, approved April 8, 1903, as to what constitutes a public building."

Section 1 provides:

"That it shall be the duty of the State Health Officer of Texas and he is hereby authorized to and is empowered to prepare rules and regulations governing the proper disinfection and sanitation of public buildings and of railway coaches and sleeping cars operated in the State of Texas."

Without undertaking to give here an exhaustive enumeration of the buildings embraced by this act, I will say that the words, "public buildings," as therein used include:

First—All buildings owned by the State or any county or incorporated city or town and devoted to governmental purposes, such as the State capitol, courthouses, city halls, jails, penitentiaries, eleemosynary institutions, etc.

Second—All buildings, whether so owned or owned privately, which are devoted to uses of a public character in contradiction to uses which are of a private nature, such as hospitals, school buildings, lecture halls, railroad depots, club rooms, opera houses, rooms for the exhibition of moving pictures, office buildings, hotels, restaurants, etc.

The enforcement of this statute involves the exercise of the police power of the State in protection and conservation of the public health, a power which has practically no limitation save that of reasonableness, to be determined and declared by the courts upon the facts of particular cases as they arise, rather than by any invariable and general rule. The courts usually construe such statutes very liberally in order to give effect to the humane purposes of the law and to enable the officers who are charged with the duty of its enforcement to effectively carry out its provisions.

FOUR REASONS WHY.

NEW YORK CITY, March 26, 1907.

*Dr. F. E. Daniel, Austin, Texas.*

DEAR SIR: More of our standardized specialties are prescribed in Texas than in any other State in the Union. This proves three things:

First—The physicians of Texas *practice* the ethics they preach. They prescribe *ethical* products. They appreciate and *demand* quality.

Second—Our ethical and pharmacal standards are bona fide. S. & D. quality means something. It is not a glib, meaningless advertising phrase. It means—the best.

Third—Our Texas representatives, headed by the genial Cornell, have won the confidence and patronage of the most ethical physicians and progressive pharmacists.

We have always advocated purity of drug, practical pharmacy and straightforward business methods—in short, honesty. To the best of our ability we have practiced what we preached.

We are greatly increasing and improving our laboratory equipment at Baltimore, and this will enable us to produce more and we hope better products than ever.

May we not, therefore, on the basis of ethics, quality and fair prices, confidently count upon your continued specification of S. & D.? Every leading druggist in Texas can supply.

We have sent you a copy of our '07 catalogue.

Very truly yours,

SHARP & DOHME.

Fourth—It also proves the value of the "Red Back" as an advertiser. S. & D. have advertised in the "Red Back" twenty-two conscutive years. They say: "If we could advertise in only two medical journals, the TEXAS MEDICAL JOURNAL would be one of them." *Verbum sat.*—DANIEL.

